# Experimental and Theoretical Study on the False Positive of Monomethyltin Determination in Toys Based on Gastric Juice Migration

**DOI:** 10.1155/2020/1975471

**Published:** 2020-03-31

**Authors:** Lina Huang, Hanke Li, Yuhang Li, Peng Ren, Lezhou Yi, Yang Chen, Tongmei Ma

**Affiliations:** ^1^Guangzhou Customs Technology Center, Huacheng Avenue 66, Zhujiang New Town, Tianhe District, Guangzhou 510623, China; ^2^School of Chemistry and Chemical Engineering, South China University of Technology, Wushan Road 381, Tianhe District, Guangzhou 510640, China

## Abstract

**Background:**

Organic tin compounds (OTCs), a group of high-risk hazardous substances, are highly concerned in safety regulation of consumer products especially for toys because they can cause serious damage to organs after prolonged or repeated exposure. Gastric juice migration is nowadays widely implemented to assess organic tin intake from toys or food-contact materials; however, the followed up detection method using sodium tetraethylborate [NaB(Et_4_)] as a derivatization agent may directly lead to false positive of monomethyltins (MMT).

**Objective:**

In order to avoid the phenomena of false positive of MMT in the course of laboratory testing of toys, it is necessary and important to perform relative experimental and theoretical studies to reveal the cause of false positive of MMT.

**Method:**

With metal tin powder as a representative of inorganic tin which existed in real samples, it was treated with artificial gastric juice (0.07 mol/L·HCl), followed by ethyl derivatization using sodium tetraethylborate [NaB(Et)_4_] and then analyzed by gas chromatography-mass spectrometry (GC-MS) according to the procedure specified in the standard of EN 71-3:2013+A3:2018 issued by the European Committee for Standardization (CEN).

**Results:**

Without any OTCs in the starting materials, MMT false positive can be reproduced by detecting 0.56 mg/L of triethylmethyltin (TEMT) together with approximately 1000 mg/L of tetraethyltin (TeET), which is similar to real samples. Further, it is demonstrated that the detected amount of TEMT is linearly related with the added amount of NaB(Et)_4_, and that the formation of TEMT (methyl derivative) is easier than TeET (ethyl derivative) even though the ethyl group is present in a larger amount than the methyl group.

**Conclusions:**

The phenomena of MMT false positive which occurred in the laboratory testing of toys is mainly because that TEMT is highly likely to be obtained from the reaction of inorganic tin and trace level of methylation agent impurities contained in the derivatization reagent—NaB(Et)_4_. To avoid MMT false positive, it is concluded that the maximum acceptable mole ratio of methylation agent impurities contained in NaB(Et)_4_ is approximately 0.028%. This research is helpful to be aware of methylation impurities and is favorable to avoid false judgment caused by MMT false positive in routine analysis of toys.

## 1. Introduction

Organic tin compounds (OTCs), a group of organic compounds which contain at least one Sn-C bond, are widely used as plastic stabilizers in toys and other consuming products for decades [1]. However, it is reported that OTCs could produce harmful effects to fertility and unborn child, further causing genetic defects, and they are also environmentally toxic as they damage aquatic life with long-lasting effects [2]. Toxicological research indicates that OTCs can cause inactivation of enzymes, damage to nervous systems, and physiological disorders [3]; thereby, being extremely harmful to children's health if they are contained in toys. To this end, OTCs have been restricted by the European Union (EU) and other developed regions. Notably, trisubstituted OTCs, dibutyltins, and dioctyltins have been included in the list of substances of very high concern (SVHC) [4] due to their reproductive toxicity and potential carcinogenicity [5], and so far, up to 11 OTCs have been restricted by the EU Toy Safety Directive (TSD) 2009/48/EC since 2009 [6] and both the limitations of detection and quantification of the 11 OTCs can be found in EN 71-3:2013+A3:2018 issued by the European Committee for Standardization (CEN) [7]. It should be emphasized that TSD has had a huge impact on the global toy industry since coming into force due to its dramatically lowered limitations for OTCs.

However, the standardized method for determination of OTCs substantially falls behind the legislation. It was not until 2013 that the European standard for the safety of toys EN 71-3:2013 specified a gas chromatography-mass spectrometry (GC-MS) method for the determination of OTCs in toy materials [7]. GC-MS has been broadly applied for determination of OTCs since it provides favorable separation and qualitative and quantitative capabilities [8, 9]. Before GC-MS analysis can be conducted, it is necessary to perform derivatization steps to convert nonvolatile or less-volatile OTCs into corresponding volatile compounds. In this regard, sodium tetraethylborate [NaB(Et)_4_] has been most frequently used as a derivatization reagent since it is much more stable and safer to be operated compared with either boron hydride or the Grignard reagent. Moreover, NaB(Et)_4_ is more practically used for analysis of large amounts of aqueous samples and beneficial for reducing matrix interferences [10, 11]. Therefore, NaB(Et)_4_ derivatization followed by GC-MS detection has become the most popular method for determination of OTCs in various standards [12–14].

It is worth noting that EN 71-3 is the first standard to adopt the gastric juice migration model to simulate the contact condition between toys and gastric juice after children had swallowed toys, wherein gastric juice is simulated by the hydrochloric acid solution to extract soluble elements including OTCs released from toys [7]. However, our previous study clearly demonstrated [15] that when analyzing surface coating materials or clear varnish on tin or tin-plated substrates according to the standard of EN 71-3, there would always be positive findings of monomethyltin (MMT), which were determined as triethylmethyltin (TEMT) in GC-MS and were typically below 10 mg/kg. To the best of our knowledge, OTCs are barely added into the coatings at such low level which is not sufficient for being functional. Besides, at normal temperature and atmosphere, tin is stable towards air or water and natural conversion of tin to OTCs barely occurs [16]. Hence MMT is suspected to be converted from tin or tin-plated materials, which is in striking contrast with the original intention of EN 71-3 where all detected OTCs are expected to be originated from OTCs already presented in samples. The so-caused MMT false positive can be facilely confused with real MMT positive, thus leading to false judgment of final results. Although EN 71-3 has received three amendments from 2014 to 2018, the MMT false positive problem is still not resolved to a large extent in the latest EN 71-3+A3:2018 [7]. Herein, in order to provide an insight into the origin of the suspected MMT false positive, both experimental and theoretical approaches were carried out systematically, during which, 0.2 g tin powder was used to simulate tin or tin-plated substrates in real samples and treated according to the procedure specified in EN 71-3:2013+A3:2018 [7] and 0.56 mg/L of TEMT was detected thereafter, confirming the aforementioned MMT was false positive.

## 2. Experimental Section

### 2.1. GC-MS and HS-GC-MS

A model SW22 thermostatic water bath provided by Julabo GmbH (Seelbach, Germany) was employed to simulate migration process in the stomach, and a model HY-2 reciprocating horizontal shaker provided by Changzhou Aohua Instrument Co. Ltd. (Changzhou, China) was used and operated at 300 cycles per minute for derivatization and extraction according to the clause G.5.1 of Annex G of EN 71-3:2013+A3:2018 [7].

The chromatographic analysis was performed on a 7890A gas chromatograph (GC) equipped with a CTC CombiPAL autosampler (CTC Analytics, Zwingen, Switzerland) and connected to a 5975C mass selective detector (MSD) (Agilent, Palo Alto, CA, USA). The column which was used was DB-WAX (100% polyethylene glycol, 30 m × 0.25 mm × 0.25 *μ*m) provided by Agilent J&W (Santa Clara, CA, USA). The sample inlet was operated at 275°C in the splitless mode, and the injection volume for liquid was set to 2 *μ*L. The oven temperature program began at 35°C and was linearly increased to 70°C at a rate of 5°C/min and subsequently to 230°C at a rate of 30°C/min and then held at 230°C for 2 min. Helium (99.999%) was used as the carrier gas and operated at a constant flow of 1 mL/min. The experimental conditions of headspace (HS) injection, such as the column, the injection mode, the oven temperature program, and the carrier gas were the same as mentioned above. The sample was incubated at 70°C for 30 min to reach a vapor-liquid equilibrium, and both the injection needle and the sample inlet were set to 80°C and the injection volume for HS was set to 1 mL. For both liquid and HS injection, the analytes were ionized by electron impact with an ionization energy of 70 eV. Transfer line, ion source, and quadrupoles were operated at 240°C, 230°C, and 150°C, respectively. A solvent delay of 4 min was used to prevent filament damage. The MSD was operated in the scan mode with a mass range of (*m*/*z*) 45–400. ChemStation software (Version E.02.00.493, Agilent, Palo Alto, CA, USA) was employed for batch processing, data acquisition, and data analysis.

### 2.2. Standards and Reagents

Methyltin trichloride (MTTC, ≥97.0%), tripropyltin chloride (TPTC, ≥98.0%), and tributyltin chloride (TBTC, ≥98.0%) standard materials were purchased from Dr. Ehrenstorfer GmbH (Augsburg, Germany). Triethyltin chloride (TETC, ≥97.0%) standard material was purchased from Alfa Aesar (Ward Hill, MA, USA). Tetraethyltin (TeET, ≥99.0%) reagent was purchased from Fluorochem (Hadfield, UK). Metal tin powder (≥99.99%, particle size ranging from 75 *μ*m to 150 *μ*m) was purchased from Aladdin (Shanghai, China). NaB(Et)_4_ reagent was purchased from International Laboratory (San Francisco, CA, USA), Strem Chemicals (Newburyport, MA, USA), and CNW Technologies GmbH (Duesseldorf, Germany), respectively. Fuming hydrochloric acid (37%, trace metal grade) was purchased from Merck (Darmstadt, Germany). Methanol, tetrahydrofuran, and n-hexane reagents of HPLC grade were purchased from Fisher Scientific (Loughborough, UK). Glacial acetic acid of HPLC grade was purchased from TEDIA (Fairfield, OH, USA). Sodium acetate trihydrate and N, N-dimethylformamide of analytical grade were purchased from Guangzhou Chemical Reagent Factory (Guangzhou, China). All these reagents and solvents were used as received unless otherwise specified. Water used in all experiments was obtained from a Milli-Q Direct (Millipore, Billerica, MA, USA) water purification system and had a resistivity higher than 18.2 MΩ·cm.

### 2.3. Preparation of Solutions

Artificial gastric juice: 0.07 mol/L hydrochloric acid solution was used as artificial gastric juice and was prepared by sequentially diluting fuming hydrochloric acid (37%) with ultrapure water.

Solutions of OTCs: TeET solution in 1000 mg/L was prepared in n-hexane. Another TeET solution in 10000 mg/L was prepared in N, N-dimethylformamide for HS-GC-MS analysis. MTTC, TETC, TPTC, and TBTC solutions all in 1000 mg/L were prepared in methanol, respectively.

Derivatization solution: 2% (*m*/*v*) of NaB(Et)_4_ solution was prepared in ultrapure water according to the clause G.3.25 of Annex G of EN 71-3:2013+A3:2018 [7], and the solution was freshly prepared daily. And, another 16000 mg/L (eqv. 1.6% *m*/*v*) NaB(Et)_4_ solution was prepared in tetrahydrofuran and kept at 4°C in absence of light.

Buffer solution: 500 mL NaOAc/HOAc buffer solution with pH = 4.5 was prepared using 16.6 g sodium acetate trihydrate, 1.2 mL glacial acetic acid, and ultrapure water.

### 2.4. Pretreatment of Tin Powders

0.2000 g tin powder was weighed in a 25 mL conical flask, and 10 mL of artificial gastric juice (preheated to approx. 20°C) was then added; the flask was sealed and agitated for 1 h at 37 ± 2°C and stood for another 1 h at 37 ± 2°C in a thermostatic water bath. The migration solution was then filtered, and 5 mL of filtered solution was transferred into a 15 mL polypropylene tube.

Derivatization procedure: 5 mL of NaOAc/HOAc buffer solution, 0.5 mL of 2% NaB(Et)_4_ solution, and 2 mL of n-hexane were added into the tube in sequence. The tube was tightly capped and shaken on a horizontal shaker for 30 min at a frequency of 300 cycles per minute and was left to stand until the phase separation had been complete, and the n-hexane extract was used for GC-MS analysis [7]. A sample-blank test was carried out simultaneously to ensure there was no contamination of any OTCs in the entire procedure.

### 2.5. Derivatization of OTC Solutions

Derivatization of MTTC, TETC, TPTC, and TBTC (qualitative analysis): take four disposable 15 mL polypropylene tubes, and to each of them, 5 mL of 0.07 mol/L hydrochloric acid was added. Then, 1.0 mL of MTTC, TETC, TPTC, and TBTC solutions (1000 mg/L in methanol) were added to each tube separately. These solutions were derivatized using the same derivatization procedure described above and then subject to GC-MS analysis.

Derivatization of TETC (quantitative analysis): take six disposable 15 mL polypropylene tubes, and to each tube, 5 mL of 0.07 mol/L hydrochloric acid, 2 mL of TETC solution (1000 mg/L in methanol), and 5 mL of NaOAc/HOAc buffer solution were successively added. 0, 62.5, 125, 375, 625, and 875 *μ*L NaB(Et)_4_ solutions in 16000 mg/L (equivalent to 0, 1, 2, 6, 10, and 14 mg NaB(Et)_4_, respectively) were then added to different tubes, and 2 mL of n-hexane was finally added to each tube. The tubes were tightly capped and shaken on a horizontal shaker for 30 min at a frequency of 300 cycles per minute and were left to stand until the phase separation was complete, and the n-hexane extract of each tube was used for GC-MS analysis.

### 2.6. Density Functional Theory Studies

The theoretical studies based on density functional theory (DFT) were performed with the DMol^3^ package to simulate the relevant transition states [17], with the assumption that the methylation agent contained in NaB(Et)_4_ was NaB(Et)_3_Me, which competed with NaB(Et)_4_ to react with TETC. The exchange-correlation functional was PBE [18]. DNP was adopted as the electronic basis set, which was nearly equivalent to 6-31G^*∗∗*^ in Gaussian. The global atomic cutoff was set to 4.9 Å. The DSPPs method was used for the core treatment. Besides, the long-range dispersion correction suggested by Grimme [19] was involved in all calculations. The electron spin state was unrestricted, and the system was negatively charged with 1e.

## 3. Results and Discussion

### 3.1. Reproduction of MMT False Positive

**Figure 1:**
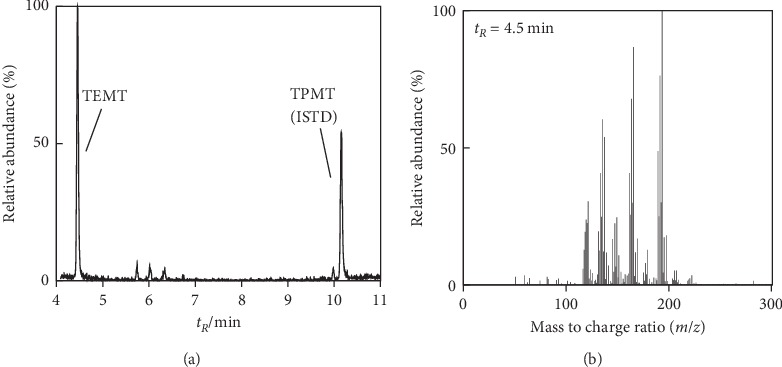
(a) Extracted ion chromatogram of ion 193 and (b) mass spectrum at *t*_*R*_ = 4.5 min. TEMT: triethylmethyltin and TPMT: tripropylmethyltin (internal standard).

Using tin or tin plating which contained OTCs-free real toy samples, our preliminary study [15] has already demonstrated that 0.44∼0.93 mg/kg MMT could be detected after treating these samples according to the procedures specified in EN 71-3+A3:2018 [7]. Here, in order to provide a better understanding, tin powder was used to simulate tin or tin-plated materials contained in toy samples and was treated by the same procedure described in Section 2.4, and the obtained n-hexane extract was then analyzed by GC-MS.  Figure 1(a) shows the extracted ion chromatogram (*m*/*z* = 193) of the extract and corresponding mass spectrum (Figure 1(b)) at 4.5 min. The mass spectrum was matched by NIST MS Search 2.2 database, giving a very high probability of 0.97 to be TEMT, and the concentration of TEMT was calculated to be 0.56 mg/L by external calibration, thus indicating elemental tin alone can be converted into TEMT.

Meanwhile, by external calibration, TeET had been detected at a level of approximately 1000 mg/L, which was much higher than that of TEMT (0.56 mg/L), indicating that TeET was the main OTC product while TEMT was one of the OTC byproducts converted from elemental tin. Moreover, different batches of NaB(Et)_4_ purchased from the International Laboratory were used for derivatization of the migration solution obtained by tin powder, and the results showed no significant difference in the concentration of TEMT. Therefore, elemental tin could consequently lead to false-positive results of MMT after being treated according to the procedure specified in Section 2, which is referred to the procedure of the standard EN 71-3:2013+A3:2018 [7].

### 3.2. Three Hypothetical Causes of MMT False Positive

After carefully examining each step of the whole testing, TEMT is most likely to be generated from two aspects, i.e., pretreatment and detection. The pretreatment aforementioned in Section 2.4 only involves one step, i.e., deriving with NaB(Et)_4_, which could possibly introduce the methyl group into the mixture; therefore, it is highly suspected that TEMT can be formed if the derivatization reagent contains any impurities with the active methyl group (e.g., NaB(Et)_3_Me). On the other hand, during detection by GC-MS, the possible decomposition of the major deriving product TeET into TEMT at high temperature can also be an issue of concern. Last but not least, the possibility of intracolumn reaction which substitutes ethyl groups of TeET with methyl groups in the stationary phase under high temperature cannot be neglected. Therefore, herein, the three most hypothetical causes of MMT false positive are proposed:

1. Cause I: TEMT was obtained from the reaction of inorganic tin and trace level of methylation agent contained in NaB(Et)_4_.
2. Cause II: TEMT was obtained from decomposition of TeET at the GC sample inlet or inside the column at high temperatures.
3. Cause III: TEMT was obtained from potential methyl-substituted reaction of TeET inside the column.

However, a survey of literature indicates that TeET is a rather stable compound and no evidence of thermal decomposition of TeET has been reported. Further, the column (DB-5MS) employed in this work is coated with a cross-linked polymer (polyethylene glycol); therefore, the likelihood of a reaction inside the column is very low. As a result, among these proposed causes, we anticipate that the first cause is most likely compared with the other two causes, and therefore detailed theoretical and experimental studies are followed with a focus on the first cause. Meanwhile, the other two clauses are also necessary to be considered thoroughly in order to figure out each possibility leading to false positive of MMT.

### 3.3. Confirmation of Cause I

Firstly, MTTC, TETC, TPTC, and TBTC standard solutions were derived for qualitative analysis. These OTCs were expected to be derived to obtain corresponding ethyl-substituted products: TEMT, TeET, TPET, and tributylethyltin (TBET); however, not only ethyl-substituted but also corresponding methyl-substituted products: diethyldimethyltin (DEDMT), TEMT, tripropylmethyltin (TPMT), and tributylmethyltin (TBMT) were detected by GC-MS, implying the presence of the methylation agent in NaB(Et)_4_.

**Figure 2:**
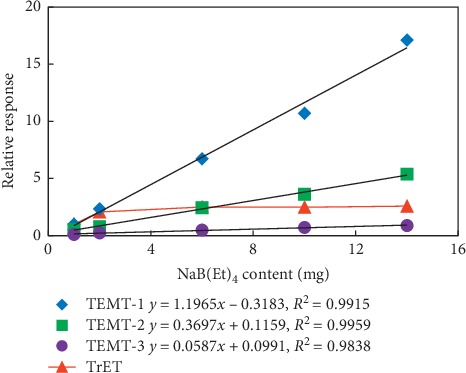
Quantitative relationship between relative responses of TEMT/TeET and amount of NaB(Et)_4_ added, where TEMT-1, TEMT-2, and TEMT-3 represent relative responses of triethylmethyltin when adding NaB(Et)_4_ provided by 3 different suppliers.

To further investigate the quantitative relationship between the amount of NaB(Et)_4_ and methyl-substituted products, TETC was chosen to react with different amount of NaB(Et)_4_. And, the results showed that when no NaB(Et)_4_ was added, no TEMT was detected, while TEMT could be detected at different concentrations when different amount (1, 2, 4, 6, 10, and 14 mg) of NaB(Et)_4_ was added. For the convenience of comparison, the instrumental response (peak area) of TEMT or TeET was set to 1 when the amount of NaB(Et)_4_ added was 1 mg, and Figure 2 illustrates the relations between the amount of TEMT (or TeET) and NaB(Et)_4_ added. Stoichiometric relationship between TETC and NaB(Et)_4_ indicated that TETC was completely consumed when 1.2 mg or more NaB(Et)_4_ was added; therefore, the instrumental response of TeET, which is the main product, will not change when more than 1.2 mg of NaB(Et)_4_ was added, which was clearly evidenced in Figure 2 that the instrumental response of TeET remained unchanged when 2 mg or more NaB(Et)_4_ was added. On the contrary, the instrumental response of TEMT increased along with increasing the amount of NaB(Et)_4_ added in a linear relationship, and the same phenomenon could be observed using different suppliers with different linearities (slope). This suggests that methylation agent impurities are commonly contained in NaB(Et)_4,_ and it was also shown that the amount of TEMT varied from different NaB(Et)_4_ suppliers. Furthermore, these results have excluded the possibility that the impurities (3% in TETC) of TETC contributed to the formation of TEMT since NaB(Et)_4_ added was in great excess.

**Figure 3:**
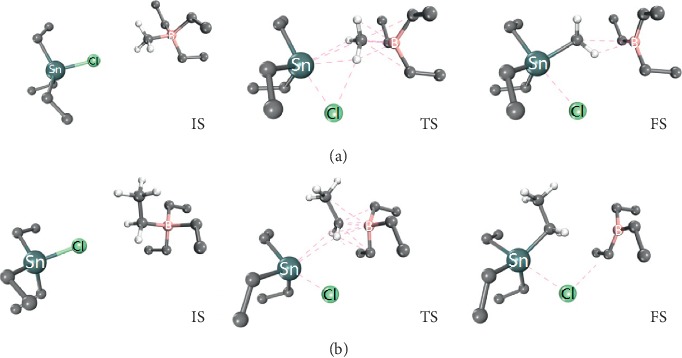
The illustration of (a) methyl substitution and (b) ethyl substitution of TETC. IS, TS, and FS stand for initial state, transition state, and final state, respectively. In the ball and stick modelling, all the unlabeled dark color balls represent carbon atoms and all the unlabeled light color balls represent hydrogen atoms. The dark green balls, light green balls, and pink balls represent tin atoms, chloride atoms, and boron atoms, respectively. Only the hydrogen atoms in the reactive alkyl radical are displayed. The dash lines mean the nonbonding weak interactions between atoms.

Theoretically, ethyl substitution of OTCs induced by NaB(Et)_4_ is known as a nucleophilic reaction in which the ethyl anion released by the tetraethylborate anion [B(Et)_4_]^−^ is a nucleophilic reagent attacking the Sn-Cl bond. Generally, according to reaction rate theory, the reaction rate of a nucleophilic reaction is dominated by the concentration of the substrate (TETC in this case) and nucleophilic reagent (ethyl and methyl anion in this case) and the reaction rate constant *k* [20], as given as follows:

(1) $$\begin{array}{c} {\text{Et}}_{3}\text{SnCl}+{\text{Nu}}^{-}={\text{Et}}_{3}\text{SnNu}+{\text{Cl}}^{-}, \end{array}$$

(2) $$\begin{array}{c} v=\frac{\text{d}\left[{\text{Et}}_{3}\text{SnCl}\right]}{\text{d}t}=k{\left[{\text{Et}}_{3}\text{SnCl}\right]}^{m}{\left[{\text{Nu}}^{-}\right]}^{n}, \end{array}$$

where Nu^−^ represents the nucleophilic reagent with one negative charge and [Et_3_SnCl] and [Nu^−^] represent the concentration of TETC and nucleophilic reagent, respectively. Noting that concentrations of the substrate, namely, TETC, were the same for both ethyl- and methyl-substitution reaction. If methylation agent impurities were contained in NaB(Et)_4_, the concentration of ethyl anions would be far greater than that of methyl anions and their ratio is independent of total amount of the alkylation reagent used. But the results showed that the methyl-substituted product depends on the total amount of the alkylation reagent used. Therefore, the reaction rate constants (*k*) of ethyl- and methyl-substituted reactions should have significant difference. According to the research conducted by Golubev et al, the reaction rate constant becomes larger with stronger nucleophilic ability of alkyl anions [21]. The coexistence of the methyl-substituted product suggested that methyl substitution might be easier to occur in comparison with ethyl substitution, or the methyl anion showed much stronger nucleophilic ability than the ethyl anion did. To confirm this, DFT studies were carried out. The methyl-substitution process of TETC is shown in Figure 3(a), where the related energy barrier is 0.587 eV and the total energy released is −0.120 eV. Similarly, Figure 3(b) represents the ethyl-substitution process of TETC, where the energy barrier is 0.766 eV and the total energy released is −0.243 eV. Note that both reactions are exergonic, illustrating that the reaction paths are thermodynamically feasible. By comparison, the energy barrier of methyl substitution is lower than that of ethyl substitution, indicating that the reaction along the former path is faster.

To sum up, the methylation agent contained in NaB(Et)_4_ directly leads to false positive of TEMT. And according to Figure 2, it is fair to say that commercially available NaB(Et)_4_ used for derivatization of OTCs commonly contains trace amount of the methylation agent, which is probably due to the impurity of raw materials. It is worth mentioning that when NaB(Et)_4_ provided by one of the suppliers (corresponding to TEMT-3 in Figure 2) was used to derive the migration solution of tin powder, only 0.038 mg/L (0.76 mg/kg) of TEMT was detected, which is much lower than the migration limit of category III toy materials specified in Directive 2009/48/EC (0.6 mg/L or 12 mg/kg) and such low level will not affect the judgment of conclusion. However, NaB(Et)_4_ provided by the other two suppliers resulted in concentrations of TEMT to be 0.56 mg/L (or 11.2 mg/kg) and 0.19 mg/L (or 3.8 mg/kg), respectively, which obviously exceeded the migration limit and thereby causing false judgments. Therefore, it is important to control the amount of methylation agent impurities contained in NaB(Et)_4_ to reduce TEMT generated in derivatization process. If 1.2 mg/kg (or 0.06 mg/L), namely, 1/10 of the migration limit (12 mg/kg), could be used as the maximum acceptable concentration of TEMT that generated from methylation agent impurities contained in NaB(Et)_4_, the maximum mole ratio (calculation based on sodium tetraethylborate) of the methylation agent was approximately 0.028% with the assumption that the methylation agent is 100% converted to TEMT. However, the amount of the methylation agent contained in NaB(Et)_4_ cannot be accurately determined by quantitative analysis of TEMT because no TEMT standard materials are commercially available for the moment.

In conclusion, the first hypothetic cause of false positive of MMT is confirmed and TEMT is obtained from the reaction of inorganic tin and trace level of methylation agent impurities contained in the derivatization reagent NaB(Et)_4_.

### 3.4. Confirmation of Cause II

**Figure 4:**
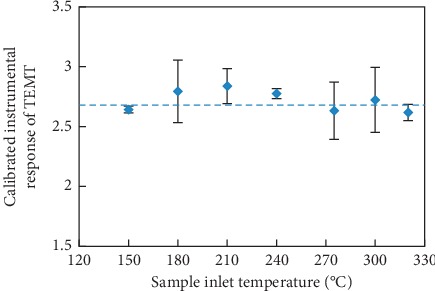
Calibrated instrumental responses of TEMT in the n-hexane extract originated from tin powder at different sample inlet temperatures (*n* = 3), where dash line represents the weighted mean of calibrated instrumental responses of TEMT calculated as in formula (3).

Although the literature survey seemingly disagrees with the second cause that TEMT was obtained from decomposition of TeET at the GC sample inlet or inside the column under high-temperature conditions, it is still necessary to consider this possibility and confirm it by experiments. By considering that a decomposition process is usually temperature dependent, the n-hexane extract of tin powder was injected to GC at different sample inlet temperatures, and the instrumental response of TEMT was monitored to investigate whether TEMT came from decomposition of TeET. It should be noticed that TEMT was not completely vaporized at low sample inlet temperatures, while as sample inlet temperature increased, more vaporized TEMT was introduced into the column, which would cause the instrumental response of TEMT to increase, hence internal standard (ISTD) calibration was applied to eliminate the effect caused by incomplete vaporization. TPMT was chosen as the internal standard to calibrate the instrumental response of TEMT since deuterated TEMT was not commercially available. The n-hexane extract was injected at 150, 180, 210, 240, 275, and 320°C, respectively, and the injection was replicated 3 times at each temperature. Instrumental responses of TEMT calibrated by TPMT at different temperatures are presented in Figure 4. To investigate whether there were significant differences of calibrated instrumental responses obtained at different sample inlet temperatures, chi-square goodness of fit was applied. The weighted means and the weighted standard deviations of calibrated instrumental responses obtained at different temperatures were calculated as formula (3), giving $\bar{R}\pm \sigma_{\bar{R}}=1.5576\;\pm \;0.008$. The corresponding chi-square value was calculated as formula (4), giving *χ*^2^=5.79, which was less than the chi-square critical value 12.69 where the confidence level was 95% and the degree of freedom was 6, meaning there were no significant difference among calibrated instrumental responses of TEMT at different sample inlet temperatures; in other words, calibrated instrumental response of TEMT was not related to the sample inlet temperature:

(3) $$\begin{array}{c} \bar{R}=\frac{\sum R_{i}/\sigma_{i}^{2}}{\sum 1/\sigma_{i}^{2}},\sigma_{\bar{R}}^{2}=\frac{1}{\sum 1/\sigma_{i}^{2}}, \end{array}$$

(4) $$\begin{array}{c} \chi^{2}=\sum_{i=1}^{7}\frac{\left(R_{i}-\bar{R}\right)}{\sigma_{i}^{2}}, \end{array}$$

where $\bar{R}$ and $\sigma_{\bar{R}}$ represent the weighted mean and the weighted standard deviation of calibrated instrumental responses of TEMT, respectively, *R*_*i*_ and *σ*_*i*_ are the arithmetic mean and the standard deviation of calibrated instrumental responses of TEMT at different sample inlet temperatures, respectively, and *χ*^2^ is the chi-square value.

**Figure 5:**
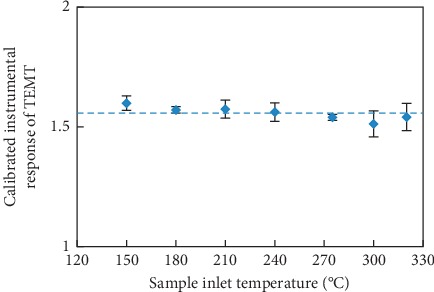
Calibrated instrumental responses of TEMT in the TeET solution at different sample inlet temperatures (*n* = 3), where dash line represents the weighted mean of calibrated instrumental responses of TEMT calculated as in formula (3).

If TEMT did not come from decomposition of TeET, TEMT should not be detected when TeET alone was analyzed by GC-MS. To confirm, 1000 mg/L of TeET solution prepared in n-hexane was directly injected to GC, and 1.26 mg/L of TEMT was then detected, indicating that TEMT could be generated from the decomposition of TeET, which conflicted with previous results. To further investigate, TeET was injected at 7 different sample temperatures as mentioned before with TPMT used as the internal standard. The instrumental responses of TEMT calibrated by TPMT at different temperatures are shown in Figure 5. Chi-square goodness of fit was also applied, giving *χ*^2^=9.34, which was also less than the chi-square critical value 12.69 where the confidence level was 95% and the degree of freedom was 6, again showing no significant difference among the calibrated instrumental responses of TEMT at different sample inlet temperatures, indicating the calibrated instrumental response of TEMT was not related to the sample inlet temperature. Therefore, TEMT was suspected to be already contained in the TeET reagent. For further confirmation, TeET solution (1000 mg/L in n-hexane) was injected to GC-MS after setting the sample inlet temperature as low as 60°C, a temperature at which decomposition hardly happened; however, TEMT could still be detected. Furthermore, HS-GC-MS analysis was performed to a saturated vapor of 10000 mg/L·TeET solution prepared in N, N-dimethylformamide according to the conditions mentioned in Section 2.3, and TEMT could still be detected; hence, it was confirmed that TEMT originally existed in the TeET reagent. In conclusion, TEMT was not generated from decomposition of TeET at the GC sample inlet under high temperature.

Since an oven temperature program was used for chromatographic analysis, if TEMT was generated from decomposition of TeET inside the column, there should be an obvious baseline ascension or a wide peak spanning for a significant period in the chromatogram for that TEMT was supposed to be gradually generated in the decomposition process. However, none of these phenomena was observed in extracted ion chromatograms of the four most abundant ions of TEMT (*m*/*z* = 193, 165, 191, and 163), and only a sharp and narrow peak was observed as shown in Figure 1 (*m*/*z* = 193), indicating that TEMT was not generated from decomposition of TeET inside the column.

In conclusion, the second hypothetic cause of false positive of MMT has been excluded, which is, TEMT is not obtained from decomposition of TeET at the GC sample inlet or inside the column under high-temperature conditions.

### 3.5. Confirmation of Cause III

Normally, a column with 5%-diphenyl-95%-dimethylpolysiloxane as a filler is preferred for separation of ethyl-derived OTCs owing to its weak polarity. However, the methyl group contained in the stationary phase might probably react with TeET to generate TEMT. Additionally, residual contamination of the sample liner could also lead to the detection of TEMT. To validate this possibility, a column free of the methyl group (100% polyethylene glycol) and a brand-new sample liner without glass wool were used. However, TEMT was still detected and its concentration was not changed, suggesting that TEMT was generated neither from methyl-substituted reaction of TeET inside the column nor from residual contamination of sample liners, thus eliminating the possibility of the third cause of MMT false positive with the conclusion that TEMT is not obtained from the potential methyl-substituted reaction of TeET inside the column.

## 4. Conclusion

In order to provide an insight into the origin of MMT false positive when analyzing tin or tin plating contained toys, both experimental and theoretical approaches were carried out. Experiments have showed that MMT false positive can be facilely reproduced by starting with elemental tin according to the procedure specified in EN 71-3:2013+A3:2018. Three possible causes of MMT false positive were proposed, and through detailed experimental and theoretical studies, it has been concluded that MMT false positive in detection of toy materials is caused by methylation agent impurities contained in the commercial derivatization agent—NaB(Et)_4_, and in order to avoid false positive judgment of MMT, the mole ratio of methylation agent impurities contained in NaB(Et)_4_ cannot be higher than 0.028%. It has also been proven in this work that TEMT is not generated by decomposition of TeET at the sample inlet or inside the column or methyl-substitution of TeET inside the column and residual contamination of the sample liner.

To avoid impact of false judgment of OTCs in routine testing of toys which contain tin or tin-plated materials, without a better method for analyzing OTCs proposed in short term, methylation agent impurities should be made aware by testing laboratories and extra attention should be drawn to NaB(Et)_4_ used for derivatization of OTCs.

## Data Availability

The figure data and related data used to support the findings of this study are included within the article.
